
JOURNAL INFORMATION
==============================
NLM Title Abbreviation: ScientificWorldJournal
Journal ID: ScientificWorldJournal
NLM Journal ID: 101131163
Journal ID: TSWJ

The Scientific World Journal

ISSN: 2356-6140
EISSN: 1537-744X
Publisher: Wiley

ARTICLE INFORMATION
==============================
PMCID: PMC5956405
PMID: 15349520
DOI: 10.1100/tsw.2004.36
Article ID: 856254
Article version: 1
Subjects: Original Article

The Molecular Basis for IntersexualityPart One: The Developing Testis

Aaronson Ian A.
Medical University of South Carolina, Charleston, SC, USA
Academic Editor: Anthony Atala

Publication date: 2004
Electronic publication date: 2004 Jun 7
Volume: 4
First page: 1
Last page: 8
Copyright: Copyright © 2004 Ian A. Aaronson.
Copyright year: 2004
License: This is an open access article distributed under the Creative Commons Attribution License, which permits unrestricted use, distribution, and reproduction in any medium, provided the original work is properly cited.
License URL: https://creativecommons.org/licenses/by/3.0/

==============================
Original Article
*TheScientificWorldJOURNAL* (2004) 4 (S1), 1–8
ISSN 1537-744X; DOI 10.1100/tsw.2004.36

### The Molecular Basis for Intersexuality

### Part One: The Developing Testis

Ian A. Aaronson, MA, MB, BChir, FRCS
*Medical University of South Carolina, Charleston, South Carolina*

*Previously published in the Digital Urology Journal*

---

**DOMAIN**: urology

---

Intersex disorders are the result of an adverse event which occurs at some stage along the pathway of normal sexual differentiation. We owe our understanding of this pathway largely to the pioneering intrauterine experiments carried out by Alfred Jost[1] in wartime France during the 1940's. He showed that by removing the gonadal ridges in the early rabbit embryo before the stage of sexual differentiation both the internal and external genitalia went on to develop along female lines irrespective of the genetic sex. In a second series of experiments, he inserted a crystal of testosterone where the ridges had been and observed that the external genitalia in all cases masculinized although the Mullerian ducts persisted and went on to form fallopian tubes and a uterus.

Jost concluded from these studies that in sexual differentiation female was the pathway of default but for normal masculinization to occur, the testes must secrete, in addition to testosterone, another substance which was responsible for suppression of the Mullerian ducts which he called ìlíhormone inhibitrice.î We now know this as Mullerian inhibiting substance or anti-Mullerian hormone (AMH), a TGF-beta- like growth factor which is produced by the Sertoli cells early in testicular development. We have subsequently come to appreciate that testosterone, produced by the Leydig cells, is converted in the peripheral tissues by the enzyme 5 alpha reductase to dihydrotestosterone which is actually responsible for the masculinizing process and that the effectiveness of these hormones depends on the integrity of testosterone receptors in the target cells. Nonetheless, it is clear that the testis occupies a central role in the pathway of male sexual differentiation.

The gonads in the early embryo are identical in both sexes and initially appear as swellings in the central portion of the urogenital ridges on either side of the dorsal midline. The first signs of testicular organization can be recognized at 42 days when cells in the medial portion of the mesonephros aggregate into cords of primordial Sertoli cells which come to envelope inflowing germ cells to form primordial tubules. Leydig cells migrate into the developing testicular interstitium where they come to rest as clusters between the tubules.[2] These events are clearly genetically determined but the precise mechanisms responsible for initiating these steps in testicular development were, until recently, unknown.

### THE Y CHROMOSOME

In the first decades of this century it was recognized that in insects the number or size of the chromosomes differed according to the sex. The realization that the fruitfly Drosophila possessed a Y

chromosome led to speculation that this was responsible for male sexual differentiation. However, breeding experiments subsequently showed that the Y chromosome was not relevant and that sex was determined by the dosage of X chromosomes, a mechanism also operative in the primitive nematode worm Caenorhabditis.[3] As sex determination is highly evolutionarily conserved, it was assumed that the situation in man would be similar.

It was, though, by the study of individuals with anomalous sexual development, particularly those with a 45X karyotype whose genitalia were uniformly female, that refocused attention on the potential importance of the Y chromosome.[4] It was, however, only with the recognition that patients with Klinefelter syndrome who have a 47 XXY karyotype, invariably had testes, and were unequivocally male[5] that it was fully understood that in man the number of X chromosomes was not relevant to male sexual differentiation. The subsequent identification of other patients with 48 XXXY and 49 XXXXY karyotypes who also had testes and a male phenotype clearly confirmed this.

The Y chromosome is only one-third of the size of the X chromosome. Its short arm (Yp) contains 13 megabase pairs of DNA whereas the long arm (Yq) contains 46 megabase pairs, but because these form numerous repetitive sequences the Y chromosome was until very recently regarded largely as a genetic wasteland.[6] During cell division, the autosomes line up in pairs each making contact with its mate thereby exchanging some genetic material. Although the X and Y chromosomes are unpaired, they also make contact with each other although usually only at the tips of their long and short arms. These regions were therefore called the pseudoautosomal regions and were thought unlikely to harbor the unique sequences required for sex determination.

In 1966, a landmark observation was made by Jacobs and Ross.[7] They described two sisters who had female external genitalia but a 46 XY karyotype in which the Y chromosome consisted only of its long arm. It seemed probable, therefore, that the testicular determining region of the Y chromosome normally resides in its short arm. This was confirmed by high resolution banding studies of an XX male which revealed that Yp material had been translocated to one of his X chromosomes.[8] This was subsequently shown to be common among XX males and provides a straightforward explanation as to why these individuals have testes. The search for the specific gene responsible for male sex determination, now termed the Testis Determining Factor, was under way.

### H-Y ANTIGEN

In the 1950's, it had been observed that when female mice were grafted with skin from isogenic males it was rejected. This indicated the Y chromosome probably contained a histocompatibility gene, which became known as the histocompatibility -Y-antigen. Wachtel[9] proposed this had a role in sex determination and reported finding expression of this gene in some XX males, although in others it seems to be absent. It was, however, only with the demonstration that XX male mice could have testes in spite of the absence of any detectable HY antigen[10] that is was finally accepted that H-Y antigen was not the testis determining factor.

### ZFY GENE

By the 1980's, the development of molecular techniques allowed gene deletions to be studied in detail. By careful comparison of nucleotide sequences in XX males and XY females it was now possible to localize the testicular determining region to the distal portion of Yp immediately adjacent to the pseudoautosomal boundary.[11] Here, a 140 Kb interval, designated ZFY, was identified which encoded a protein with multiple zinc ìfingerî domains. This seemed to be a strong candidate for the testis determining factor but it was puzzling that ZFY was also present on the X chromosome and, moreover, that it was expressed in many other tissues besides the testis. The hypothesis that this was the gene for testicular determination had to be abandoned when XX phenotypic males were identified whose X chromosomes contained translocated Y material which was devoid FZY.[12]

### SRY GENE

The search for the testis determining factor ended in 1990 when Sinclair,[13] also examining the translocated material in XX males, identified a 35 Kb sequence in the Yp 11.3 band immediately adjacent to the pseudoautosomal boundary which was identical to that found in normal males. This was confirmed to be evolutionarily conserved and appeared to be the only gene on the Y chromosome necessary for the development of the testis. It was named the Sex determining Region of the Y chromosome (SRY).

Support for the designation of SRY as the testis determining factor came from the study of mouse embryos where, in the genital ridge SRY was found to be expressed for only a very short period immediately proceeding the first appearance of the Sertoli cells. Furthermore, its expression could not be found in any other embryonic tissues and it was therefore testis specific. Conclusive evidence came from an elegant experiment in which a 14 Kb DNA fragment containing SRY but no other functioning genes was inserted into the genome of female mice embryos who, as a result, developed testes and external genitalia which were male.[14]

The SRY gene is now known to comprise a single exon within which lie 237 base pairs which encode an evolutionarily highly conserved group of 79 aminoacids known as the HMG box, so called because of their homology to the widely distributed Highly Mobile Group of proteins. Collectively, this family of genes, 14 of which have so far been identified, has been named the SRY HMG box or ìSOXî gene family.

The SRY gene acts as a transcription factor. Its protein product recognizes a specific DNA sequence AACAAT to which it becomes bound.[15] A non-polar side chain first inserts into a minor groove of the DNA double helix which then undergoes an acute bend of approximately 80 degrees[16], resulting in widening of the groove and partial unwinding of the DNA helix.[17] This special rearrangement of the double helix brings into close approximation regions of DNA on either side of the SRY target site, thus potentially making available other sites to which further transcription factors might bind, although these have yet to be identified. The critical importance of the HMG box can be seen in patients with point mutations or microdeletions in this region of the SRY gene where a single aminoacid substitution can lead to profound alterations in the ability of the protein to bind or bend DNA.[18]

### OTHER TESTICULAR PATHWAY GENES

Although it is now clear that the SRY gene plays a central role in triggering the formation of the testis two puzzling groups of patients indicate that other genes, located either on the X chromosome or on autosomes, must also be involved in testicular differentiation. Firstly, although some XY females have mutations or deletions in their SRY gene which explain why they do not have testes, in approximately 85% of such cases the SRY gene appears to be normal. Furthermore, although the majority of XX males have a translocation of their SRY gene, in approximately 20% of cases this cannot be detected. It is also known that in several clinical syndromes resulting from autosomal deletions or mutations testicular development is impaired. It is by studying these patients that further light has been shed on the testicular developmental pathway.

### WT-1 GENE

Among patients with the WAGR (Wilmsí tumor, Aniridia, Genital malformations and mental Retardation) syndrome the testes are frequently dysgenetic with a propensity to form gonadoblastomas. Gonadal dysgenesis and genital ambiguity are also features of the related Denys-Drash syndrome which, in addition to Wilmsí tumor, is characterized by an early onset protein losing nephropathy. In these syndromes deletions have been detected in the WT-1 tumor suppressor gene located in the chromosome 11p13 band.[19]

These clinical observations focused attention on the role of WT-1 in testicular development. Examination of human embryos has revealed that WT-1 is expressed not only, as expected, in the developing kidney but also in the primitive gonad indicating that here too it plays a role.[20] This is supported by studies of mice bred with a null mutation of the WT-1 gene. It was found that embryos homozygous for this deletion had in addition to bilateral renal agenesis complete absence of ovaries or testes. This indicated that the WT-1 tumor suppressor gene is essential early on in the formation of the indifferent gonad from the intermediate mesoderm[21], thus placing WT-1 upstream of SRY in the testicular developmental cascade.

### SF-1

Steroidogenic factor 1 (SF-1) is a nuclear receptor protein, the gene for which has been mapped to chromosome 9. It regulates the production of the hydroxylase enzymes necessary for the synthesis of steroids and is therefore expressed in many tissues throughout the body including the adrenal gland, ovary and Leydig cells of the testis. It was a surprise, therefore, when SF-1 transcripts were detected in the mouse urogenital ridge at the stage of the indifferent gonad before the Leydig cells had appeared.[22] This suggested that in addition to its steroidogenic role, SF-1, is also an early player in the testicular developmental cascade.

In order to test this hypothesis, mice were bred with a deletion for the fushi tarazu factor 1 (Ftz-F1) gene, which is structurally very similar to SF-1. At birth the animals appeared normal but within a few days all died from adrenal insufficiency. At autopsy, all were found to be completely lacking not only in adrenal glands but also in gonads, thus confirming the pivotal role of SF-1 in early gonadal development.[23]

Within the testis SF-1 acts not only to establish Leydig cell testosterone biosynthesis but is also expressed in the Sertoli cells where it appears to act as a regulator of AMH production.[24] The SRY gene also seems to have a regulatory effect on AMH production but whether SRY and SF-1 themselves interact in some way is presently not clear.[25]

### SOX-9

Among the SOX family of genes, SOX-9, which has been assigned to chromosome 17, appears to be a candidate for a role in testicular development. Mutations of this gene are routinely found in patients of both sexes with campomelic dysplasia, a dominantly inherited and usually fatal osteochondrodysplasia.[26] Among those with an XY karyotype approximately 75% also have dysgenetic gonads which give rise to ambiguous genitalia which is a characteristic feature of this condition.

Studies of normal human fetuses have shown SOX-9 to be expressed, as expected, in chondrocytes but also in the differentiated testis indicating that it has a role relatively late in the developmental pathway.[27] In addition a recent report suggests that, at least in the mouse, SOX-9 is also expressed early in the genital ridge before any gonadal differentiation has occurred.[28] Its precise role at these sites is presently unclear although it may possibly be concerned with the formation of connective tissue upon which the integrated architecture of both the bones and the testes depends.

### DAX-1 GENE

In 1980, Bernstein[29] reported a pair of female siblings with dysgenetic gonads who had a 46 XY karyotype in which a portion of the short arm of the X chromosome was duplicated. This raised the question whether suppression of their testes was the result of a double dose of X material, reminiscent of the sex determining mechanism in the fruitfly and nematode. Other cases have subsequently been identified in whom the SRY gene was intact but who, in addition, had an X fragment of 160 kilobases duplicating part of the Xp21 band.[30] This was named the Dosage Sensitive Sex reversal region of the X (DSS) which overlaps a locus where deletions are known to occur in patients with adrenal hypoplasia congenita. The gene responsible for sex reversal in these cases was therefore designated DAX-1 (for DSS-AHC critical region of X) which is now known to encode a nuclear hormone receptor protein.[31]

Until very recently it was doubtful that DAX-1 played any role in the development of the testes for, although cryptorchidism is sometimes seen with adrenal hypoplasia congenita, these patients invariably have testes and normal male external genitalia. It was thought to have a role in ovarian development but the recent finding that DAX-1 is expressed in the mouse genital ridge[32] suggests that, as well as adrenal development, this gene may be important early on in the formation of the indifferent gonad.

### OTHER AUTOSOMAL GENES:

Evidence for the involvement of other genes in the development and differentiation of the gonads comes from several case reports. Bennett[33] described a 46XY female with gonadal dysgenesis in whom a terminal deletion was found in the short arm of chromosome 9. Another XY patient with ambiguous genitalia and testicular dysgenesis was reported by Wilkie[34] in whom a deletion was noted in the long arm of chromosome 10. Gonadal dysgenesis with subsequent phenotypic ambiguity is also a feature of a number of disorders including the Smith-Lemli-Opitz syndrome, the genito-palato-cardiac, and the alpha thalassemia and mental retardation syndromes.

### CONCLUSIONS

Over the past 50 years our understanding of the role of the Y chromosome in determining the sex of the embryo has progressively evolved from initial doubt as to its importance to an appreciation of its key role which came in 1990 with the cloning of the SRY gene. Since then we have learned how the SRY gene product recognizes, binds to and distorts its target DNA, a process which most likely initiates yet more steps in the cascade, although precisely what these are remains to be discovered. What has become clear is it that SRY is not the only gene necessary to form a testis (see Table). We now know that the WT-1 and SF-1 genes, as well as possibly SOX-9 and DAX-1 are also needed for the early induction of the indifferent gonad. Furthermore, it is becoming apparent that several of these genes act not only at more than one site in the developmental cascade but also in more than one organ system in the body.[35] The figure summarizes the steps presently known to be involved in the development and differentiation of a functioning testis (see Figure).

Hitherto, our assumption has been that all of the above genes act as transcription activators, each triggering in a positive way the next step along a cascade. Yet several paradoxes remain. How is it possible, for example, that some XX males seem to be totally lacking in SRY, yet the majority of XY females seem to be amply endowed with this gene? To explain these puzzles McElreavy[36] proposed that the SRY gene product acts not as an activator but primarily as a repressor of another hitherto unidentified gene ìZî the role of which is to inhibit testis formation. This, and other models are currently being explored to explain the remaining clinical and experimental puzzles. Although tantalizingly near, we are still a little way from finally understanding the molecular complexities of testicular development.

**TABLE**
**Known or candidate genes in the testicular developmental cascade.**

| Chromosome | Locus | Gene |
|---|---|---|
| Y Chromosome | p11.3 | SRY |
| X Chromosome | p21-p22 | DAX-1 |
| Chromosome 9 | q33 | SF-1 |
| Chromosome 11 | p13 | WT-1 |
| Chromosome 17 | q24-q25 | SOX-9 |
| Chromosome 19 | p13.3 | AMH |

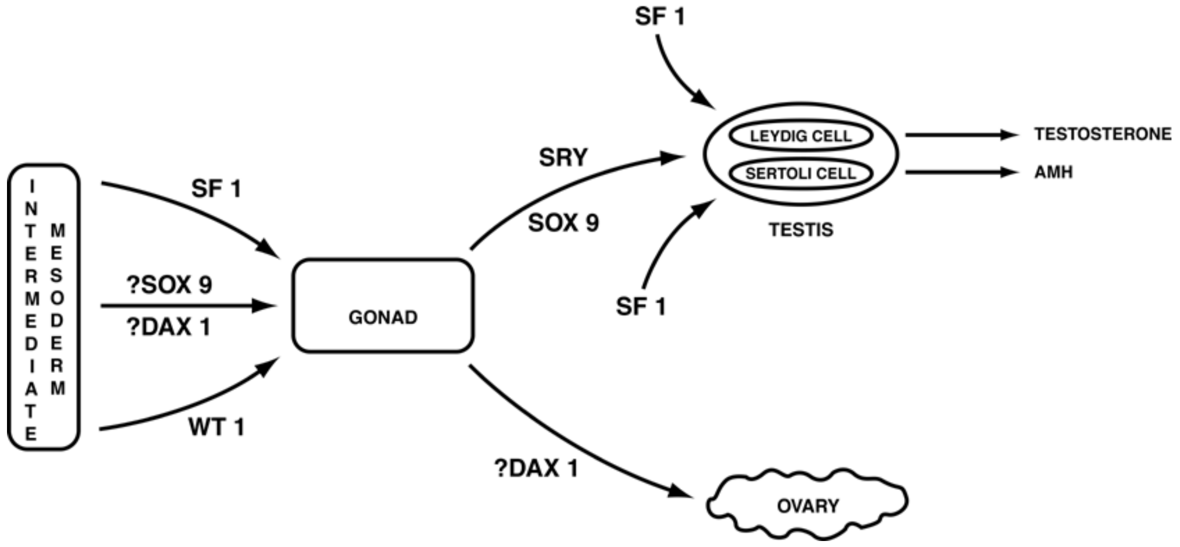

**FIGURE 1.** Proposed pathway of gonadal development and differentiation.

### REFERENCES

1. Jost, A. Recherches sur la differenciation sexuelle de líembryon de lapin. III. Role des gonades foetales dans la differenciation somatique. Arch. Anat. Microsc. Morphol. Exp. 36: 271-316, 1947.
2. Capel, B. The role of Sry in cellular events underlying mammalian sex determination. Current Topics in Developmental Biology 32: 1-37, 1996.
3. Nicoll, M., Akerib, C.C., and Meyer, B.J. X-chromosome-counting mechanisms that determine nematode sex. Nature 388: 200-204, 1997.

4. Ford, C.E., Jones, K.W., Miller, O.J., Mittwoch, U., Penrose, L.S., Ridler, M., and Shapiro, A. The chromosomes in a patient showing both mongolism and the Klinefelter syndrome. Lancet 1: 709-710, 1959.
5. Jacobs, P.A., and Strong, J.A. A case of human intersexuality having a possible XXY sex determining mechanism. Nature 183: 302-303, 1959.
6. Lahn, B.T., and Page, D.C. Functional coherence of the human Y chromosome. Science 278: 675-680, 1997.
7. Jacobs, P.A., and Ross, A. Structural abnormalities of the Y chromosome in man. Nature 210: 352-354, 1966.
8. Madan, K. Chromosome measurements on a XXp+ male. Hum. Genet. 32: 141-142, 1976.
9. Wachtel, S.S., Ohno, S., Koo, G.C., and Boyse, E.A. Possible role for H-Y antigen in the primary determination of sex. Nature 257: 235-236, 1975.
10. McLaren, A. Sex determination in mammals. Trend Genet. 4: 153-157, 1988.
11. Page, D.C., Mosher, R., Simpson, E.M., Fisher, E.M., Mardon, G., Pollack, J., McGillivray, B., de la Chapelle, A., and Brown, L.G. The sex determining region of the human Y chromosome encodes a finger protein. Cell 51: 1091-1104, 1987.
12. Palmer, M.S., Sinclair, A.H., Berta, P., Ellis, N.A., Goodfellow, P.N., Abbas, N.E., and Fellous, M. Genetic evidence that ZFY is not the testis-determining factor. Nature 242:937-939, 1989.
13. Sinclair, A.H., Berta, P., Palmer, M.S., Hawkins, J.R., Griffiths, B.L., Smith, M.J., Foster, J.W., Frischauf, A.M., Lovell-Badge, R., and Goodfellow, P.N. A gene from the human sex determining region encodes a protein with homology to a conserved DNA binding motif. Nature 346: 240-244, 1990.
14. Koopman, P., Gubbay, J., Vivian, N., Goodfellow, P., and Lovell-Badge, R. Male development of chromosomally female mice transgenic for Sry. Nature 351: 117-121, 1991.
15. Harley, V.R., Lovell-Badge, R., and Goodfellow, P.N. Definition of a consensus DNA binding site for SRY. Nucleic Acids Res. 22: 1500-1501, 1994.
16. Ferrari, S., Harley, V.R., Pontiggia, A., Goodfellow, P.N., Lovell-Badge, R., and Bianchi, M.E., SRY, like HMG 1, recognizes sharp angles in DNA. EMBO J. 11: 4497-4506, 1992.
17. Werner, M.H., Huth, J.R., Gronenborn, A.M., and Clore, G.M. Molecular basis for human 46XY sex reversal revealed from the three dimensional solution structure of the human SRY-DNA complex. Cell 81: 705-714, 1995.
18. Pontiggia, A., Rimini, R., Harley, V.R., Lovell-Badge, R., and Goodfellow, P.N. Sex reversing mutations affect the architecture of SRY-DNA complexes. EMBO 13: 6115-6124, 1994.
19. Mueller, R.F., The Denys-Drash syndrome. J. Med. Genet. 31: 471-477, 1994.
20. Pritchard-Jones, K., Fleming, S., Davidson, D., Bickmore, W., Porteous, D., Gosden, C., Bard, J., Buckler, A., Pelletier, J., Houseman, D., van Heyningen, V., and Hastie, N. The candidate Wilmsí tumour gene is involved in genitourinary development. Nature 346:194-197, 1990.
21. Kreidberg, J.A., Sariola, H., Loring, J.M., Maeda, M., Pelletier, J., Houseman, D., and Jaenisch, R. WT-1 is required for early kidney development. Cell 74: 679-691, 1993.
22. Ikeda, Y., Shen, W.H., Ingraham., H.A., and Parker, J.L. Developmental expression of mouse steroidogenic factor-1, an essential regulator of the steroid hydroxylases. Mol. Endocrinol. 8: 654-662, 1994.
23. Luo, X., Ikeda, Y., and Parker, K.L. A cell specific nuclear receptor is essential for adrenal and gonadal development and sexual differentiation. Cell 77: 481-490, 1994.
24. Shen, W.H., Moore, C.C., Ikeda, Y., Parker, K.L., and Ingraham, H.A. Nuclear receptor steroidogenic factor 1 regulates the Mullerian inhibiting substance gene: A link to the sex determination cascade. Cell 77: 651-661, 1994.
25. Schafer, A.J., and Goodfellow, P.N. Sex determination in humans. BioEssays 18: 955-963, 1996.
26. Meyer, J., Sudbeck, P., Held, M., Wagner, T., Schmitz, M.L., Brickarelli, F.D., Eggermont, E., Friedrich, U., Haas, O.A., Kobelt, A., Leroy, J.G., Van Maldergem, L., Michel, E., Mitulla, B., Pfeiffer, R.A., Schinzel, A., Schmidt, H., and Scherer, G. Mutational analysis of the SOX 9 gene in campomelic dysplasia and autosomal sex reversal: lack of genotype/phenotype correlations. Hum Mol Genet. 6: 91-98, 1997.
27. Wagner, T., Wirth, J., Meyer, J., Zabel, B., Held, M., Zimmer, J., Pasantes, J., Bricarelli, F.D., Kentel, J., Hustert, E., Wolf, U., Tommerup, N., Schempp, W., and Scherer, G. Autosomal sex reversal and campomelic dysplasia are caused by mutations in and around the SRY-related gene SOX 9. Cell 79: 1111-1120, 1994.
28. Kent, J., Wheatley, S.C., Andrews, J.E., Sinclair, A.H., and Koopman, P. A male-specific role for SOX-9 in vertebrate sex determination. Development 122: 2813-2822, 1996.
29. Bernstein, R., Koo, G.C., and Wachtel, S.S. Abnormality of the X chromosome in Human 46 XY female siblings with dysgenetic ovaries. Science 207: 768-769, 1980.
30. Bardoni, B., Zanaria, E., Guioli, S., Floridia, G., Worley, K.C., Tonini, G., Ferrante, E., Chiumello, G., McCabe, E.R., Fraccaro, M., Zuffardi, O., and Camerino, G. A dose sensitive locus at chromosome Xp21 is involved in male to female sex reversal. Nature Genet. 7: 497-501, 1994.
31. Zanaria, E., Muscatelli, F., Bardoni, B., Strom, T.M., Guioli, S., Guo, W., Lalli, E., Moser, C., Walker, A.P., McCabe, E.R., Meitinger, T., Monaco, A.P., Sassone-Corsi, P., and Camerino, G. An unusual member of the nuclear hormone receptor superfamily responsible for X-linked adrenal hypoplasia congenita. Nature 372: 635-641, 1994.
32. Swain., A. Zanaria, E., Hacker, A., Lovell-Badge, R., and Camerino, G. Mouse DAX-1 expression is consistent with a role in sex determination as well as in adrenal and hypothalamus function. Nature Genet.12: 404-409, 1996.
33. Bennett, C.P., Docherty, Z., Robb, S.A., Ramani, P., Hawkins, J.R., and Grant, D. Deletion 9p and sex reversal. J. Med. Genet. 30: 518-520, 1993.

34. Wilkie, A.O., Campbell, F.M., Daubeney, P., Grant, D.B., Daniels, R.J., Mullarkey, M., Alfara, N.A., Fitchett, M., and Huson, S.M. Complete and partial XY sex reversal associated with terminal deletion of 10q: Report of 2 cases and literature review. Am. J. Med. Genet. 46:597-600, 1993.
35. Graves, J.A. Two uses for old SOX. Nature Genet. 16: 114-115, 1997.
36. McElreavey, K., Vilain, E., Abbas, N., Herskowitz, I., and Fellous, M. A regulatory cascade hypothesis for mammalian sex determination: SRY represses a negative regulator of male development. Proc. Natl. Acad. Sci. USA 90: 3368-3372, 1993.

**This article should be referenced as follows:**

Aaronson, I.A. (2004) The molecular basis for intersexuality. Part one: the developing testis. *TheScientificWorldJOURNAL* **4 (S1)**, 1–8.

**Handling Editor:**

Anthony Atala, Principle Editor for *Urology* — a domain of *TheScientificWorldJOURNAL*.
